# Superior Stone

**Published:** 1859-10

**Authors:** 


### SUPERIOR STONE.

Dr. Robbins, of Meadville, Pa., at the late meeting of the Convention, presented us a specimen of polishing stone, obtained by him in the Lake Superior regions, which we find, in many respects, far better than the famous Arkansas stone. It puts a very fine finish on gold, and does not clog in the least with the metal. It is reduced to the desired shape much more readily than the Arkansas stone, and shows less brittleness when reduced to a thin edge. Altogether, we like it extremely, and hope it will be kept for sale at all the dental depots.

Dr. R. has our hearty thanks for the favor. W.

